# Variability at the 3′ untranslated region of the *HLA-G* gene: a study on patients with AIDS and cytomegalovirus retinochoroiditis

**DOI:** 10.1038/s41598-020-75639-9

**Published:** 2020-10-29

**Authors:** Luciana de Morais Vicente, Erick C. Castelli, Maria de Lourdes Veronese Rodrigues, Neifi Hassan Saloum Deghaide, Celso Teixeira Mendes-Junior, João M. Furtado, Eduardo Antonio Donadi

**Affiliations:** 1 Ophthalmology Division, Departament of Ophthalmology, Otorhinolaryngology and Head and Neck Surgery, Ribeirão Preto Medical School, University of São Paulo, Av. Bandeirantes, 3900, Ribeirão Preto, SP 14049-900 Brazil; 2 Pathology Department, School of Medicine, São Paulo State University (UNESP), Botucatu, SP Brazil; 3 Departamento de Química, Faculdade de Filosofia, Ciências e Letras de Ribeirão Preto, Universidade de São Paulo, Ribeirão Preto, SP Brazil; 4 Clinical Immunology Division, Department of Internal Medicine, Ribeirão Preto Medical School, University of São Paulo, Ribeirão Preto, SP Brazil

**Keywords:** Immunology, Molecular biology, Diseases

## Abstract

Cytomegalovirus retinochoroiditis (CMV-R) is the primary cause of blindness among AIDS patients. Since HLA-G is associated with the modulation of the immune response, we hypothesized that variability at the 3′ untraslated region (3′UTR) of the gene could be implicated on the predisposition to CMV-R. We evaluated whether *HLA-G* 3′UTR influences CMV-R development in Brazilian AIDS patients. Peripheral blood DNA was obtained from two groups of patients: (1) AIDS exhibiting CMV-R (n = 40) and (2) AIDS without CMV-R (n = 147). *HLA-G* 3′UTR typing was performed using sequencing analysis. Allele, genotype and haplotype frequencies were evaluated using Fisher’s exact test accompanied by the calculation of the odds ratio and its 95% confidence interval (95% CI). The etiologic (EF) and preventive fractions were also estimated. Compared to AIDS patients without CMV-R, AIDS patients with CMV-R showed increased frequencies of the: (1) + 3001T allele, (2) the + 3001C/T genotype and (3) the UTR-17 (InsTTCCGTGACG) haplotype (EFs = 0.02–0.04). The UTR-3 (DelTCCGCGACG) haplotype was associated with protection against CMV-R development. Although the risk for developing CMR-V at the population level was relatively low (EF), the identification of *HLA-G* 3′UTR variation sites may help to further evaluate the role of post-transcriptional factors that may contribute to the existent immunosuppresion caused by HIV per se.

## Introduction

Cytomegalovirus retinochoroiditis (CMV-R) primarily affects patients with AIDS exhibiting CD4 cell counts < 50 cells/mm^3^. Although the frequency of CMV-R has been decreased due to the universal access of patients with AIDS to the combination antiretroviral therapy (cART), retinochoroiditis continues to be an important cause of severe visual loss, particularly in countries where cART is not freely available^1^. Thus, the identification of AIDS patients prone to develop CMV-R, the early diagnosis and early treatment may propiciate a reduction on the number of years of blindness in patients whose survival is favored by cART^2^.

Considering that *HLA-G* has a well recognized immunodulatory role, inhibiting the cytotoxic effects of CD8 and natural killer (NK) lymphocytes^3^, there is a clear possibility of exploring this gene in the search for innovative immunoregulatory strategies for diagnostic and therapeutic strategies in various conditions, among them AIDS and CMV-R.

Advances in gene sequencing have shown several variation sites along the *HLA-G* coding and regulatory regions, which may affect protein diversity and gene expression. Variation sites at the *HLA-G* promoter or 3′ untranslated region (3′UTR) may affect transcriptional and post-transcriptional gene control by modifying target sites for transcription factors and microRNAs, respectively^4^. In the Brazilian population, at least 37 single nucleotide polymorphisms (SNP) at the promoter^5^ and at least 16 variation sites at 3′UTR have been described for the *HLA-G* gene^6^.

Several polymorphisms at the *HLA-G* 3′UTR segment can influence gene expression^7^. The insertion/deletion (Ins/Del) of 14 base pairs is the best-characterized polymorphism described at 3′UTR, which has been associated with mRNA stability^8–10^. The + 3142 G/C SNP has also been reported to post-transcriptionally control HLA-G expression, since the presence of a Guanine may increase the affinity of the primary mRNA for the microRNAs (miR)-148a, miR-148b and miR-152^5–11^. The + 3187 A/G polymorphic site is located at the proximity of an AU-rich motif, which also affects mRNA stability^12^. Little information is available regarding other variation sites at *HLA-G* 3′UTR, including + 3001T/C, + 3003T/C, + 3010 G/C, + 3027 C/A, + 3035 C/T and + 3196 C/G, which are potential binding sites for miRNAs, and thus may post-transcriptionally regulate HLA-G expression^13–15^. Since the variation sites at the *HLA-G* 3′UTR are in linkage disequilibrium, several 3′UTR haplotypes have been described, of which at least 15 haplotypes are observed in Brazilians^5,16^.

Although studies regarding the classical HLA class I and class II profiles of patients with AIDS exhibiting ocular manifestations have been reported so far^17–19^, there is no information regarding the study of the non-classical *HLA-G* gene in patients exhibiting both AIDS and CMV-R. The present study was undertaken based on the hypothesis that variation sites at the *HLA-G* 3′UTR in patients with AIDS exhibiting or not CMV-R may discriminate variation sites associated with the regulation of the immune checkpoint gene.

## Results

*HLA-G* 3′UTR variability was assigned on the basis of the following polymorphic sites, including: 14bpINS/DEL (rs371194629) and eight variable sites: + 3001C/T (rs567747015), + 3003C/T (rs1707), + 3010C/G (rs1710), + 3027A/C (rs17179101), + 3035C/T (17179108), + 3142C/G (rs1063320), + 3187A/G (9380142), + 3196C/G (rs1610696).

The frequency of the *HLA-G* 3′UTR alleles is shown in Table 1. Only the + 3001T allele was overrepresented in CMV-R AIDS patients compared to AIDS without CMV-R (*P* = 0.046), conferring an OR 18.631 (95% CI 0.885–392.043) and an Etiologic Fraction = 0.024.

**Table 1:** Frequency and absolute number of the *HLA-G* 3′UTR alleles observed in patients with AIDS exhibiting CMV retinitis (CMV-R: Group I) and in patients with AIDS without CMV-R (Group II).

*HLA-G* position	Allele	Group I (N = 40)	Group II (N = 147)	Odds ratio	Etiologic fraction	*P* value
+ 2960 (14 bp)	Ins	0.3750 (30)	0.3741 (110)			
	Del	0.6250 (50)	0.6259 (184)			
+ 3001C/T	C	0.9750 (78)	1.0000 (292)			
	T	0.0250 (2)	0.0000 (0)	18.631 (95% CI 0.885–392.043)	0.024	0.046
+ 3003C/T	C	0.1625 (13)	0.1054 (31)			
	T	0.8375 (67)	0.8946 (263)			
+ 3010C/G	C	0.4625 (37)	0.5510 (162)			
	G	0.5375 (43)	0.4490 (132)			
+ 3027C/A	C	0.9750 (78)	0.9524 (280)			
	A	0.0250 (2)	0.0476 (14)			
+ 3032C/G	C	0.0125 (1)	0.0034 (1)			
	G	0.9875 (79)	0.9966 (293)			
+ 3035C/T	C	0.8875 (71)	0.8810 (259)			
	T	0.1125 (9)	0.1190 (35)			
+ 3142C/G	C	0.5385 (42)	0.4490 (132)			
	G	0.4615 (36)	0.5510 (162)			
+ 3187A/G	A	0.7250 (58)	0.6837 (201)			
	G	0.2750 (22)	0.3163 (93)			
+ 3196C/G	C	0.7179 (56)	0.7109 (209)			
	G	0.2821 (22)	0.2891 (85)			
+ 3227A/G	A	0.0405 (3)	0.0429 (6)			
	G	0.9595 (71)	0.9571 (134)			

The *HLA-G* 3′UTR genotype frequencies are shown in Table 2. The + 3001CC was underrepresented in CMV-R AIDS patients when compared to patients with AIDS without CMV-R (*P* = 0.045, OR 0.053, 95% CI 0.002–1.117) conferring a Preventive Fraction = 0.945, whereas the + 3001CT was overrepresented in same comparison (*P* = 0.045, OR 19.026, 95% CI 0.895–404.579, conferring an Etiologic Fraction = 0.047).

**Table 2:** Frequency and absolute number of the *HLA-G* 3′UTR genotypes observed in patients with AIDS exhibiting CMV retinitis (CMV-R: Group I) and in patients with AIDS without CMV-R (Group II).

*HLA-G *position	Genotype	Group I (N = 40)	Group II (N = 147)	Odds ratio	Etiologic fraction	Preventive fraction	*P* value
+ 2960 (14bp)	DD	0.3750 (15)	0.3946 (58)				
	DI	0.5000 (20)	0.4626 (68)				
	II	0.1250 (5)	0.1429 (21)				
+ 3001C/T	CC	0.9500(38)	1.0000(146)	0.053 (95% CI 0.002–1.117)	–	0.945	0.045
	CT	0.0500 (2)	0.0000 (0)	19.026 (95% CI 0.895–404.579)	0.047	–	0.045
	TT	0.0000 (0)	0.0000 (0)				
+ 3003C/T	CC	0.0250 (1)	0.0068 (1)				
	CT	0.2750 (11)	0.1973 (29)				
	TT	0.7000 (28)	0.7959 (117)				
+ 3010C/G	CC	0.2000 (8)	0.2857 (42)				
	CG	0.5250 (21)	0.5306 (78)				
	GG	0.2750 (11)	0.1837(27)				
+ 3027C/A	AA	0.0000 (0)	0.0000 (0)				
	AC	0.0500 (2)	0.0952 (14)				
	CC	0.9500 (38)	0.9048 (133)				
+ 3032C/G	CC	0.0000 (0)	0.0000 (0)				
	CG	0.0250 (1)	0.0068 (1)				
	GG	0.9750 (39)	0.9932 (146)				
+ 3035C/T	CC	0.7750 (31)	0.7687 (113)				
	CT	0.2250 (9)	0.2245 (33)				
	TT	0.0000 (0)	0.0068 (1)				
+ 3142C/G	CC	0.2821(11)	0.1837 (27)				
	CG	0.5128 (20)	0.5306 (78)				
	GG	0.2051 (8)	0.2857 (42)				
+ 3187A/G	AA	0.4750 (19)	0.4354 (64)				
	AG	0.5000 (20)	0.4966 (73)				
	GG	0.0250(1)	0.0680 (10)				
+ 3196C/G	CC	0.4359 (17)	0.4762 (70)				
	CG	0.5641 (22)	0.4694 (69)				
	GG	0.0000 (0)	0.0544 (8)				
+ 3227A/G	AA	0,0000 (0)	0.0000 (0)				
	GA	0.0811 (3)	0.0857 (6)				
	GG	0.9189 (43)	0.9143 (64)				

Regarding the 3′UTR haplotypes, the frequency of the UTR-17 (InsTTCCGTGACG) haplotype is higher in patients with AIDS who developed CMV-R compared with patients with AIDS without CMV-R (*P* = 0.046), conferring an OR 18.557 (95% CI 0.881–390.713) and an Etiologic Fraction = 0.025. In contrast, the UTR-3 (DelTCCGCGACG) haplotype was underrepresented in CMV-R patients when compared to controls (*P* = 0.014), conferring an OR 0.285 (95% CI 0.099–0.820) and a Preventive Fraction = 0.116 (Table 3).

**Table 3:** Frequency and absolute number of the *HLA-G* 3′UTR haplotypes observed in patients with AIDS exhibiting CMV retinitis (CMV-R: Group I) and in patients with AIDS without CMV-R (Group II).

*HLA-G *haplotype	2960 (14 bp)	3001	3003	3010	3027	3032	3035	3142	3187	3196	3227	Group 1	Group II	Odds ratio, etiologic fraction (EF) or preventive fraction (PF)	*P* value
UTR-1	DEL	C	T	G	C	G	C	C	G	C	G	0.2632 (20)	0.3007 (83)		
UTR-2	INS	C	T	C	C	G	C	G	A	G	G	0.2368 (18)	0.2500 (69)		
UTR-3	DEL	C	T	C	C	G	C	G	A	C	G	0.0526 (4)	0.1630 45)	OR = 0.285 (95% CI 0.099–0.820) PF = 0.116	0.014
UTR-4	DEL	C	C	G	C	G	C	C	A	C	G	0.1579 (12)	0.0978 (27)		
UTR-5	INS	C	T	C	C	G	T	G	A	C	G	0.0658 (5)	0.0616 (17)		
UTR-6	DEL	C	T	G	C	G	C	C	A	C	G	0.0789 (6)	0.0290 (8)		
UTR-7	INS	C	T	C	A	G	T	G	A	C	G	0.0132 (1)	0.0507 (14)		
UTR-10	DEL	C	T	C	C	G	C	G	A	G	G	0.0395 (3)	0.0217 (6)		
UTR-13	DEL	C	T	C	C	G	T	G	A	C	G	0.0000 (0)	0.0036 (1)		
UTR-15	INS	C	T	C	C	G	C	G	A	C	G	0.0263 (2)	0.0036 (1)		
UTR-17	INS	T	T	C	C	G	T	G	A	C	G	0.0263 (2)	0.0000 (0)	OR = 18.557 (95%CI 0.881–390.713) EF = 0.025	0.046
UTR-18	DEL	C	T	G	C	G	C	C	A	C	A	0.0263 (2)	0.0072 (2)		
UTR-49	DEL	C	T	G	C	G	C	C	G	G	G	0.0000 (0)	0.0072 (2)		
UTR-50	INS	C	T	C	C	G	C	G	G	G	G	0.0132 (1)	0.0036 (1)		

## Discussion

Due to the systemic immunodepression, patients with AIDS are susceptible to opportunistic pathogens, and one of the most frequent is CMV that, in approximately 85% of cases, induces CMV-R, particularly in patients presenting other risk factors (e.g. CD_4_^+^ cells count < 50). Despite the broad availability of cART in many countries, 4.9–7.6% of the patients are believed to develop CMV-R during some evolution stages of AIDS^20–24^.

The individual variability regarding the time of onset and the rate of progression of CMV-R suggest that genetic factors such as histocompatibility alleles and *HLA* specific haplotypes may be involved in disease susceptibility. The study of genetic susceptibility in infectious diseases usually poses a challenge because most studies compare infected patients with healthy controls, for whom one cannot predict whether or not the individual would be susceptible to or resistant to the infectious agent. In this study, both groups were infected by HIV and developed AIDS, and just one developed CMV-R. Noteworthy, the CMV infection is present in 90% of the healthy Brazilian individuals^24^ and in about 98% of the patients with AIDS^25^. Because the previous contact with CMV in Brazilian patients with AIDS is extremely frequent, the development of CMV-R was the dependable variable.

Considering that low CD4 cell count itself propitiate an immunosuppressive condition to develop CMV-R^17^, and also considering that HLA-G has immunosuppressive properties, the study of the regulatory region of the *HLA-G* gene is of relevance to predict the differential chance of ocular involvement in patients with AIDS. No literature data have been reported regarding the variability at the 3′UTR of the *HLA-G* gene in patients with AIDS, exhibiting or not CMV-R. Noteworthy, in the present series, just one variable site was associated with CMV-R when this group of AIDS patients was compared with AIDS patients without CMV-R. Accordingly, the + 3001T allele, the + 3001C/T genotype, and the UTR-17 (InsTTCCGTGACG) haplotype contains the + 3001T allele, and they were all associated with susceptibility to CMV-R. The + 3001C/T variable site is uncommon in healthy Brazilian individuals, but has been encountered in African populations^24^. This allele was also associated with infectious diseases, including leprosy^4^.

Although some polymorphic sites at the *HLA-G* 3′UTR (14 bp Ins/Del, + 3142C/G and + 3187A/G) have been functionally studied^11,13^, the + 3001C/T has not, and in analogy to other variation sites it may be a differential target for microRNA binding. In addition to the low frequency of + 3001C/T, the etiologic fractions conferred by the + 3001T allele, + 3001C/T genotype and UTR-17 haplotype were also low, varying from 0.02 to 0.04, thus contributing to 2–4% of the CMV-R susceptibility at the population level. This result indicates that the + 3001C/T variation may act in concert with other variable sites at *HLA-G* 3′UTR or surrounding variants. Indeed, the + 3001T allele is in linkage disequilibrium with the 14 bp Insertion, + 3142G and the + 3187A alleles, which are included at the *HLA-G* UTR-17 haplotype, and these variable sites have individually been associated with decreased production of HLA-G^7,11,13^.

Considering the 5′UTR segment, UTR-17 is associated with the *HLA-G* promoter PROMO-0103e (0103 group), which contains variation sites that diverge from the most common promoter haplotypes (Promo-010101 and PROMO-010102 groups), particularly at positions − 725 (a T nucleotide compared to C or G nucleotides in other haplotypes), − 509 (a G nucleotide compared to a C nucleotide in other haplotypes) and − 56 (a T nucleotide compared to a C nucleotide in other haplotypes)^5^. In contrast to susceptibility alleles, the + 3001C/C and the UTR-3 haplotype (exhibiting the + 3001C allele) were more robustly associated with protection against CMV-R, conferring a preventive fraction of 0.945 and 0.116, respectively. Previous studies from our group have reported that UTR-3 has been associated with median levels of *HLA-G* production^26^. Concluding, considering that the rare + 3001T allele appeared in few patients with AIDS and CMV-R, and considering the large variation of the confidence interval including the number 1, only the study of a large sample of patients would ascertain the role of the + 3001T allele on the susceptibility to CMV-R in patients with AIDS.

Besides *HLA-G*, other genes of the Major Histocompatibility Complex have been previously associated with CMV-R, including: (1) the overrepresentation of *TNF* microsatellites (*TNFc2* alleles, as well as the 4-1-G-2-2-1 haplotype, which contains the *TNFc2* allele) in patients with AIDS, inducing a rapid progression of the disease as well as the development of accelerated CMV-R^27^, (2) the HLA-A31 antigen was associated with the development of AIDS, irrespective of the presence or not of CMV-R^17^, (3) the HLA-C*07 allele group was assoaciated with protection against the development of CMV-R when compared to patients with AIDS without CMV-R. In addition, the HLA-C*05 allele group was associated with susceptibility to and the HLA-C*16 allele group with protection against AIDS development, irrespective of the presence or not of CMV-R^19^, and (4) the HLA-B35 antigen was associated with the rapid progression of AIDS and development of retinochoroidites induced by *Toxoplasma gondii*^18^.

The classical HLA class I molecules (HLA-A, -B and -C) play a major role on the defense against viruses by presenting virus-derived peptides to cytotoxic TCD8 cells, and the lack of classical class I molecules on the surface of virus infected cells provides a signal for the cytotoxic action of NK cells. In this aspect, HLA-G may inhibit the cytotoxic activity of both CD8 and NK cells, facilitating virus escape from the host immune response and permitting virus spread^6,28^. Although *HLA-A, HLA-B* and *HLA-C* form common extended haplotypes due to the linkage disequilibrium among these genes, the most striking linkage disequilibrium of the *HLA-G* gene is with the *HLA-A* gene^29,30^, and the typing of both classical and non-clasical class I genes is important to understand the association of HLA genes with virus disorders.

In this study, we reported that susceptibility to or protection against CMV-R in patients with AIDS is associated with specific variation sites at the *HLA-G* 3′UTR, and these findings deserve further studies to evaluate specific post-transcriptional factors that may target these gene segments, and consequently the differential production of HLA-G. The genetic susceptibility to produce differential levels of immunomodulatory molecules such as HLA-G, in concert with the immunosuppression caused by the HIV infection, may further influence host immunosurveillance, propitiating the concomitant appearance of other viral infections, including CMV-induced complications.

## Methods

All investigation was performed following the guidelines of the National Committee of Ethics in Research (CONEP) Brazil. The study was submitted to the Ethics Committee of the University Hospital of the Ribeirão Preto Medical School, demanding the use of samples stored at a Bank of Biological Samples (protocols # 1525/1998, #8992/2001 and #7581/2007). The Ethics Committee approved the use of the stored samples, waiving the informed consent (protocol #4084/2009). All additional patients who were further included in the study signed informed consent.

The samples were obtained from two groups of individuals: Group I (n = 40) patients with AIDS and CMV-R diagnosed according to current criteria; and Group II (n = 147), patients with AIDS diagnosed according to current criteria and without CMV-R diagnosis, regardless of the evolutionary phase of the latter or of whether or not they had started antiviral treatment.

DNA was obtained using a salting-out procedure. The 3′UTR of the *HLA-G* gene was amplified by the polymerase chain reaction (PCR) using with two primers: HLAG8R—GTCTTCCATTTATTTTGTCTCT and HLAG8F—TGTGAAACAGCTGCCCTGTGT in a final volume of 25 μL, containing ultrapure deionized water and amplification buffer (0.2 M Tris–HCl, 0.5 M KCl, pH 8.5), 0.2 mM DNTP, 1.50 mM d MgCl_2_, 5 pmol of each primer, 0.5 U DNA polymerase Platinum (INVITROGEN, Carlsbad, CA, USA), and 200 ng genomic DNA. PCR-conditions consisted of an initial cycle at 94 °C for 5 min followed by 30 cycles of three stages: 95 °C for 45 s, 56 °C for 45 s and 72 °C for 1 min, followed by a final stage at 72 °C for 7 min. The final product of the amplification reaction was sequenced (Genetic Analyzer ABI PRISM 310 (Applied Biosystems, Foster City, CA, USA) with the HLAG8R—GTCTTCCATTTATTTTGTCTCT primer using the BIGDYE Terminator v3.1 device (Applied Biosystems, Foster City, CA, USA).

*HLA-G* 3′UTR alleles and genotypes were estimated by direct counting using the GENEPOP 3.4 software^31^. Linkage disequilibrium was evaluated using the ARLEQUIN v.3.1^32^ software and adherence to Hardy–Weinberg equilibrium was determined by the Guo and Thompson test^33^ using the GENEPOP 3.4 software^31^. Since the base sequencing in the gamete phase was unknown, the haplotypes were inferred using the PHASE^34^ and Expectation-Maximization^35^ methods.

The allele, genotype and haplotype frequencies were compared between groups by Fisher’s exact test with the calculation of the Odds Ratio (OR) and 95% confidence interval (95% CI). The level of significance was set at *P* ≤ 0.05. We also estimated the etiologic fraction that indicates the contribution of each allele, genotype, or haplotype to the susceptibility to CMV-R, and the preventive fraction, that indicates how much these factors contribute to protection against the development of CMV-R.
